# Retroperitoneoscopic adrenalectomy may be superior to laparoscopic transperitoneal adrenalectomy in terms of costs and profit: a retrospective pair-matched cohort analysis

**DOI:** 10.1007/s00464-023-10395-1

**Published:** 2023-09-01

**Authors:** Andreas Fischer, Oliver Schöffski, Anna Nießen, Alexander Hamm, Ewan A. Langan, Markus W. Büchler, Franck Billmann

**Affiliations:** 1 Department of General, Visceral and Transplantation Surgery, University Hospital Heidelberg, Im Neuenheimer Feld 420, 69120 Heidelberg, Germany; 2 Fachbereich Wirtschaftswissenschaften, Lehrstuhl für Gesundheitsmanagement, Friedrich-Alexander-University Erlangen-Nürnberg, Lange Gasse 20, 90403 Nürnberg, Germany; 3 Department of Dermatology, University Hospital Schleswig Holstein, Campus Lübeck, Ratzeburger Allee 160, 23538 Lübeck, Germany; 4 Department of Dermatological Science, University of Manchester, Manchester, UK

**Keywords:** Adrenalectomy, Minimally invasive surgical procedures, Benefits and costs, Multivariate analysis

## Abstract

**Background:**

A direct comparison of the cost–benefit analysis of retroperitoneoscopic adrenalectomy (RPA) versus the minimally invasive transperitoneal access (LTA) approach is currently lacking. We hypothesized that RPA is more cost effective than LTA; promising significant savings for the healthcare system in an era of ever more limited resources.

**Methods:**

We performed a monocentric retrospective observational cohort study based on data from our Endocrine Surgery Registry. Patients who were operated upon between 2019 and 2022 were included. After pair-matching, both cohorts (RPA vs. LTA) were compared for perioperative variables and treatment costs (process cost calculation), revenue and profit.

**Results:**

Two homogenous cohorts of 43 patients each (RPA vs. LTA) were identified following matching. Patient characteristics between the cohorts were comparable. In terms of both treatment-associated costs and profit, the RPA procedure was superior to LTA (costs: US$5789.99 for RPA vs. US$6617.75 for LTA, *P* = 0.043; profit: US$1235.59 for RPA vs. US$653.33 for LTA, *P* = 0.027). The duration of inpatient treatment and comorbidities significantly influenced the cost of treatment and the overall profit.

**Conclusions:**

RPA appears not only to offer benefits over LTA in terms of perioperative morbidity and length of hospital stay, but also has a superior financial cost/benefit profile.

**Graphical abstract:**

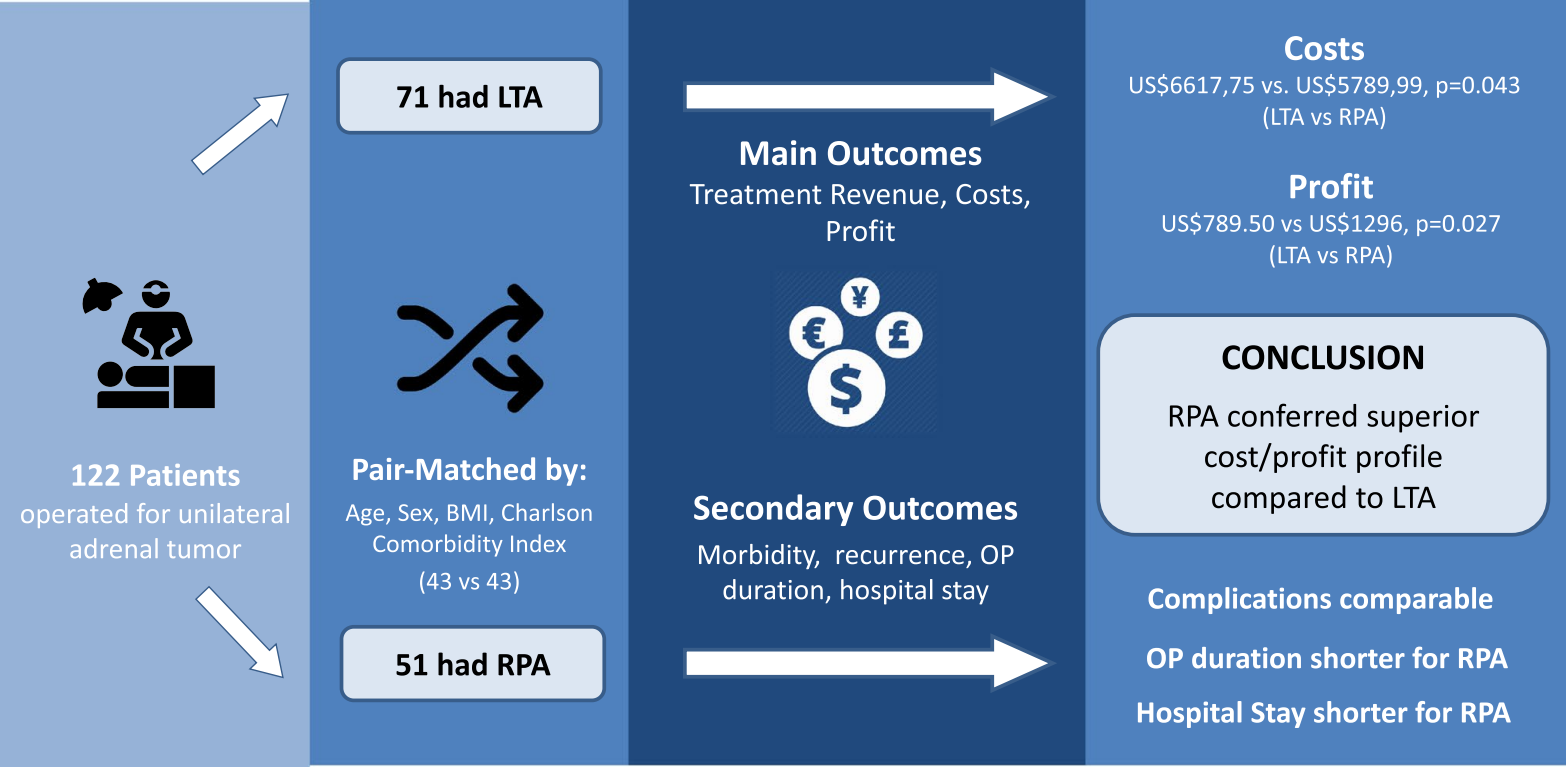

**Supplementary Information:**

The online version contains supplementary material available at 10.1007/s00464-023-10395-1.

Health care costs have risen dramatically in recent decades and now account for a significant proportion of the global gross domestic product (GDP) (17% of the country’s GDP in the United States or Europe) [1]. In Germany, gross value added (GVA) in the core sector of the health care amounted to almost €441 billion in 2020, more than 13.1% of the country’s GDP [2]. With an annual growth rate of 3.3%, the health sector has grown significantly faster than GDP over the past decade [3] with surgical care accounting for almost one third of healthcare expenditure [4, 5]. As surgically treatable diseases are expected to account for two thirds of the global lost years by 2025 [6], efforts to investigate and control costs would be wise to focus on surgical care, particularly in the area of minimally invasive surgery, where highly specialized tools may potentially lead to higher costs than traditional open surgery.

However, economic studies attempting to ascertain the cost/benefit profile of surgical interventions face several challenges, including the absence of benchmarks (e.g. costs of care in the operating room, costs per time in the radiology) and a lack of consensus of what contributes to the overall costs [7]. This is further complicated by a lack of published literature on which cost estimation models are most suitable [4, 8–15]. While some of the operative and perioperative costs associated with retroperitoneoscopic adrenalectomy (RPA) and the minimally invasive transperitoneal access approach (LTA) have been reported [16], a comprehensive cost–benefit analysis directly comparing both procedures is lacking. Moreover, the published literature does not address revenue and profit.

Meta-analyses proved RPA to be associated with lower long-term complications, a shorter hospital stay and duration of surgery and less postoperative pain [16–22]. Therefore, the aim of our study was to test the hypothesis that RPA is less expensive and eventually leads to more profit than the standard minimally invasive method (LTA) employing a global, process-oriented assessment of costs, revenue and profit.

## Patients and methods

### Study design and patients

This was a retrospective monocentric observational cohort study. All patients included in this study underwent minimally invasive adrenalectomy without robotic assistance. Patient, procedure and follow-up data were prospectively collected and maintained in our Registry for Endocrine Surgery between 2012 and 2022 (Department of General, Visceral and Transplant Surgery). In order to ensure consistent data, only patients treated between 2019 and 2022 were investigated. Preoperative screening and assessment of patients was performed by a multidisciplinary team, consisting of endocrine surgeons, endocrinologists, internists and radiologists, all with experience in assessing patients before and after adrenal surgery. Our investigation has been reported in line with the CHEERS and STROCSS criteria (Supplementary Files 1, 2) [23, 24].

### Patients and surgical technique

All patients were operated on by experienced and certified endocrine surgeons (DGAV Certification: Reference Center for Endocrine Surgery) [25]. The minimally invasive surgical techniques (LTA and RPA) which were adopted complied with guidelines of the American Association of Endocrine Surgeons (AAES) [26], the German Association of Endocrine Surgeons (CAEK) [27] and Society of the American Gastrointestinal and Endoscopic Surgeons (SAGES) [28]. These techniques have been described previously [29–31]. The choice of surgical technique was left to the patient, after having been informed of the surgical details of the technique and after having ensured that both techniques were applicable.

## Methods

The comparability of the two groups (LTA vs. RPA) in terms of perioperative variables was tested after matching (see “Statistical analysis” section). Two aspects were taken into account to test our hypothesis: (1) the calculation of the costs incurred by both surgical techniques and (2) the calculation of the revenue/profit.

### Perioperative patient characteristics

The evaluation of clinical, laboratory and imaging patient data was performed retrospectively using the above mentioned database. Operative time was measured from incision to skin closure. Postoperative complications were coded according to the Clavien–Dindo classification [32].

### Calculation of the revenue

The revenue calculation for each patient was performed retrospectively using the German Diagnosis Related Groups (gDRG) codes. These codes represent a classification system to match financial reimbursement according to each inpatient case. gDRG covers all operational costs incurred during the stay. The calculation of the revenue (*R*) was based for each patient on its relative treatment weight and the gDRG base rate, according to the formula [33]:

$$R = {\text{Relative weight}} \times {\text{Base rate}}$$

Deductions and supplements may apply for both groups (RPA vs. LTA). Two calculation methods were used: (1) the patient gDRG masks were grouped in the hospital information system (ISH med® system, SAP SE, Dietmar-Hopp-Allee 16, 69190 Walldorf, Germany) and calculated retrospectively; (2) as a control, a query was carried out in our accounting system. Both values were examined for plausibility and compared for each patient.

### Calculation of the costs

A process cost calculation was used (health economic analysis plan). That calculation was structured in three stages: (1) process analysis, (2) identification of process variables (cost drivers) and (3) cost analysis [34, 35]. The process analysis was carried out on the basis of a personal survey of the cost center managers: (1) the nursing department, (2) the lead surgeon, (3) the lead anesthetist, (4) the radiologist, (5) a physiotherapist, (6) the head of the laboratory medicine department, (7) the lead pharmacist and (8) the commercial head of the surgical clinic. The process variables (cost drivers) (e.g. case numbers, number of patients, length of stay or duration of treatment [35]) were defined and evaluated (using InEK cost matrix [33]). Resource utilization was then used to calculate the process cost rates. The full cost recalculation could than been carried out. Adjustment were made for inflation. The individual costs were allocated directly to the patients on the basis of the documented consumption of medical goods (e.g. medicines, surgical instruments, surgery time). In the allocation of overhead costs, the case-related cost allocation was carried out via a process or reference calculation. The calculation rates were calculated on the basis of suitable reference quantities, such as the PPR minutes for the personnel costs of the nursing service for the normal ward.

### Calculation of the profit

After revenue and cost analysis, a profit analysis was carried out. Profit was defined as the difference between revenue and costs. The profit was calculated for each individual case (patient), so that both positive and negative profit values could be calculated. The profit was calculated on a case-by-case basis, so that both positive and negative profit values were calculated. Subsequently, a statistical comparison between the two procedures (RPA vs. LTA) was performed.

### Statistical analysis

Graphpad Prism 9 software, version 9. 3. 1 (350) (Graphpad Software, 2365 Northside Dr. Suite 560, San Diego, CA, 92108) was used for statistical analysis. Statistical advice was provided by the biostatistics department of our university surgical clinic.

### Patient pair-matching

Pair matching was performed to be able to compare the cost, revenue and profit data among groups (RPA vs. LTA) and to minimize confounding bias. The surgical procedure used (RPA vs. LTA) was recorded as a dependent variable in the matching model and age (± 5 years), gender, body mass index BMI (± 5 kg/m^2^), presence of comorbidities (Charlson Comorbidity Index, CCI) (≤ 1 point) as independent covariates [36]. Tumors up to 4 cm in diameter were operated on in our series. Studies have shown that the laterality and size of adrenal tumors (for tumors < 6 cm) did not affect the duration of surgery or postoperative morbidity, either for LTA or RPA. Therefore, these two variables were not included as independent variables in our matching [37–39].

### Analysis of endpoint variables

The normality of the distribution was investigated with the Kolmogorov Smirnov test. Normally distributed quantitative variables were described using the mean with standard deviation (SD), and not normally distributed quantitative variables using the median with interquartile range (IQR). Categorical variables were described in absolute numbers and percentages. Differences between groups were analyzed for quantitative normally distributed variables with a one-way ANOVA test, with a Mann–Whitney *U* test for quantitative non-normally distributed variables and with *χ*^2^ or Fischer’s Exact test for categorical variables. In order to investigate the significant influence parameters on the costs and revenue of both surgical procedures, a multiple logistic regression analysis was performed. Variables supposed to affect costs and profit entering multiple logistic regression were: sex (male vs. female), laterality (left vs. right), surgical technique (LTA vs. RPA), resection type (partial vs. total), tumor size (≤ 40 mm vs. > 40 mm), morbidity (yes vs. no), BMI (≤ 25 vs. > 25 kg/m^2^), hormonal activity (yes vs. no), prior abdominal surgery (yes vs. no), CCI (≤ 2 vs. > 2) and hospital stay (≤ 5 vs. > 5 days). Graphpad gives *P* values with three decimal places. *P* values < 0.05 were considered significant.

## Results

### Study flow chart

**Fig. 1:**
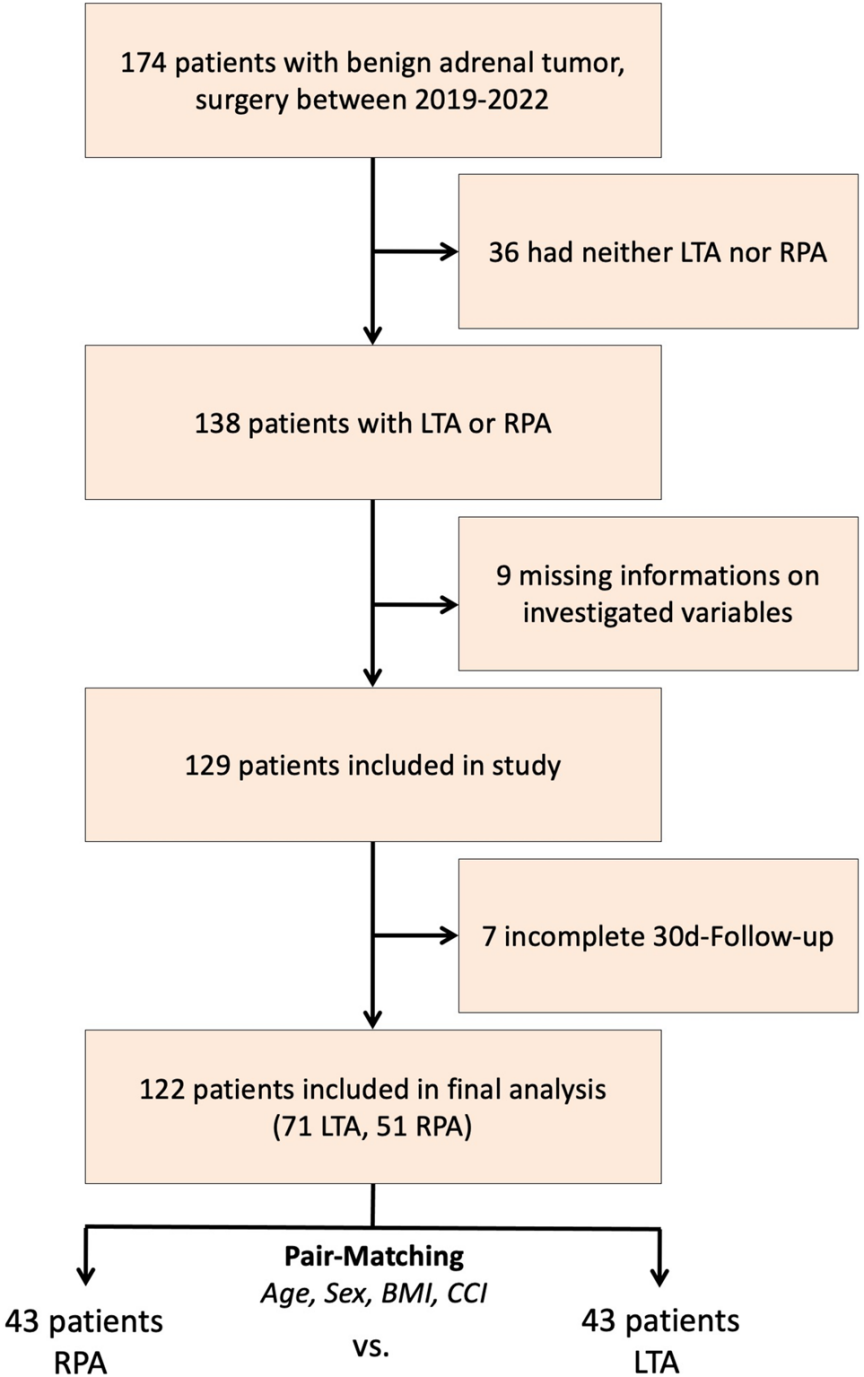
Study flowchart. *LTA* lateral transperitoneal adrenalectomy, *RPA* retroperitoneoscopic adrenalectomy

We identified a total of 174 patients who had undergone surgical treatment for a benign adrenal tumor between 2019 and 2022 in our center. 36 of these patients did open surgery and were therefore excluded from further analyses. Of the 138 remaining patients, 9 had to be withdrawn from the study due to lack of documented information on the endpoints. 7 patients were excluded as they did not complete the required 30 days of follow-up. A total of 122 patients (71 after LTA and 51 after RPA) were included in the final analysis. The flow chart summarizing patient selection is included in Fig. 1.

### Patient matching

122 patients were entered into our matching algorithm to form two comparable cohorts. Based on the covariates age, sex, BMI and CCI, our matching was able to produce two homogenous cohorts with 43 patients in each (Fig. 1). All of the following data are based on these two patient cohorts.

### Patient characteristics, intra- and postoperative outcome variables

After matching for age, sex, BMI and CCI there were no significant differences between the group in terms of histology, tumor laterality and rates of conversion to open surgery (Table 1). However, RPA patients tended to have more severe pre-existing disease than LTA patients (higher ASA score). These differences were not statistically significant (*P* = 0.353 and *P* = 0.555, respectively).

**Table 1:** Baseline patient characteristics and perioperative variables

Variable	Total (*n* = 86)	Surgical approach		*P*
		LTA (*n* = 43)	RPA (*n* = 43)	
Age (years)^a^, mean (SD), years	58.8 (9.5)	58.9 (10.0)	58.7 (9.1)	0.950
Sex ratio (M:F)	32:54	16:27	16:27	1.000
BMI (kg/m^2^), mean (SD)	24.1 (2.1)	24.0 (2.0)	24.2 (2.1)	0.719
ASA score, *n* (%)				0.353
ASA 1	2 (2.3)	2 (4.7)	0 (0.0)	
ASA 2	58 (67.4)	29 (67.4)	29 (67.4)	
ASA 3	26 (30.2)	12 (27.9)	14 (32.6)	
ASA 4	0 (0)	0 (0)	0 (0)	
Charlson Cormorbidity Index (CCI),^b^ *n* (%)				0.555
< 2	29 (33.7)	17 (39.5)	12 (27.9)	
≥ 2	57 (66.3)	26 (60.5)	31 (72.1)	
Postop. follow-up (months), median (range)	24.1 (6–49)	22.8 (9–46)	24.8 (6–49)	0.400
Operation duration (min)^c^, median (IQR)	42.0 (37.0–67.5)	67.0 (51.0–76.0)	37.0 (35.0–39.0)	< 0.0001^‡^
Estimated blood loss (mL), mean (SD)	31 (54)	33 (40)	30 (54)	0.776
Type of resection, *n* (%)				0.069
Total adrenalectomy	74 (86.0)	40 (93.0)	34 (79.1)	
Partial adrenalectomy	12 (14.0)	3 (7.0)	9 (20.9)	
Conversion, *n* (%)	0 (0.0)	0 (0.0)	0 (0.0)	1.0
Tumor laterality, *n* (%)				0.663
Left	49 (57.0)	26 (60.5)	23 (53.5)	
Right	37 (43.0)	17 (39.5)	20 (46.5)	
Histology, *n* (%)				0.164
Conn-adenoma	20 (23.3)	6 (14.0)	14 (32.6)	
Cushing-adenoma	17 (19.8)	8 (18.6)	9 (20.9)	
Pheochromocytoma^d^	19 (22.1)	12 (27.9)	7 (16.3)	
Incidentaloma/other	30 (34.8)	17 (39.5)	13 (30.2)	
Hospital stay (days), median (IQR)	5 (4–7)	5 (5–8)	5 (4–7)	0.040^‡^
Recurrence, *n* (%)	1.0	0 (0.0)	0 (0.0)	1.0

*LTA* lateral transperitoneal adrenalectomy, *RPA* retroperitoneoscopic adrenalectomy, *SD* standard deviation, *IQR* interquartile range, *BMI* body mass index, *ASA* American Society of Anaesthesiologists

^‡^Statistically significant (*P* < 0.05)

^a^At the time of operation

^b^CCI, described in [27]

^c^Operation duration: operative time was measured from incision to skin closure

^d^A specific cost analysis related to the treatment of pheochromocytoma-related complications was not performed. None of the patients included in this series received intensive care treatment. Postoperative hypertensive complications were comparable between the two treatment groups

The analysis of the intra- and postoperative course (Table 1) showed that both cohorts (LTA vs. RPA) were comparable with regard to these variables (*P* > 0.05), excepted for the average duration of the procedure (67 min for LTA vs. 37 min for RPA *P* < 0.0001) and hospital stay (6.1 vs. 4.9 days, *P* = 0.040). An evaluation of postoperative mortality between the groups revealed no significant differences. However, there was a significant difference in overall morbidity (27.9% for RPA vs. 53.5%, for LTA, *P* = 0.016) in line with the published literature [40].

### Revenue calculation

Also slightly higher in the LTA group, the mean revenues were comparable in both cohorts (*P* = 0.487). The mean revenue for LTA patients was US$7244.61 ± 472.58, whereas the RPA group presented a mean revenue of US$7204.42 ± 436.40.

### Cost calculation

The analysis in terms of total hospital treatment costs (mean ± SD; median (IQR) values, respectively) showed that the LTA procedure [US$6617.75 ± 2198.78; US$5512 (4915–9007)] was significantly more expensive than the RPA technique [US$5789.99 ± 1615.92; US$5006 (4409–7535)] (*P* = 0.043).

Leading cost centers were similar for both procedures. With a decreasing share of the total costs, there are: (1) nursing normal ward (15.78% of the total costs vs. 14.38% for RPA and LTA), (2) infrastructure normal ward (12.60% vs. 12.32%), (3) medical needs operating theater (7.77% vs. 10.84%), (4) physicians operating theater (7.07% vs. 7.38%). Table 2 compares the leading cost centers for both procedures. This analysis showed significant differences for the following cost centers (mean value in US$): (1) medical needs operating room (US$828.6 vs. 589.8 for LTA and RPA, *P* = 0.013), (2) infrastructure operating room (US$257.0 vs. 208.2, *P* = 0.025). The comparative analysis of the leading cost centers indicated that the leading share of costs is spent on medical services (22.15% vs. 22.15% for RPA and LTA), followed by infrastructure costs (20.45% vs. 20.51%), nursing services (18.87% vs. 17.61%), medical-technical services (10.95% vs. 10.29%) and medical needs (8.00% vs. 10.86%). The costs of the leading providers represented 89.53% vs. 90.04% of the total costs for the RPA and LTA procedure, respectively.

**Table 2:** Cost analysis^a^: comparison of leading cost centers LTA vs. RPA (US$)

Cost centers (US$), mean (SD)	Surgical approach		*P*
	LTA (*n* = 43)	RPA (*n* = 43)	
Physicians NW	476.1 (218.0)	592.8 (644.0)	0.881
Physicians OR	515.8 (135.5)	568.8 (274.7)	0.623
Physicians A	342.5 (60.3)	420.4 (176.2)	0.332
Nursing NW	1001.3 (545.1)	1177.9 (1548.2)	0.872
Medical needs OR	828.6 (157.4)	589.8 (336.3)	0.013^‡^
Medical infrastructure NW	269.5 (118.8)	259.8 (107.5)	0.669
Infrastructure NW	860.8 (491.7)	947.5 (770.5)	0.876
Infrastructure OR	257.0 (47.4)	208.2 (70.4)	0.025^‡^

*LTA* lateral transperitoneal adrenalectomy, *RPA* retroperitoneoscopic adrenalectomy, *NW* normal ward, *OR* operating room, *A* anesthesia

^‡^Statistically significant

^a^Cost calculation was done in US$ to allow comparison with other studies in the literature

### Profit calculation

The median profit (IQR) for the LTA procedure was US$789.50 (− 509 to 1618) and US$1296 (359 to 2426) for the RPA procedure. This difference was statistically significant (*P* = 0.027).

### Factors influencing the cost of treatment and the profit of both surgical procedures

The relationship between clinical variables (age, gender, complication, etc.) and the risk of costs > US$7500 was investigated with univariable and multivariable logistic regression. The choice of this cut-off was based on the results of the revenue analysis related to the two LTA and RPA techniques in our series. The value of US$7500 represents the cost limit at which the cost of treatment exceeds income and thus leads to a negative profit. Results are summarized in Table 3. Surgical approach (LTA vs. RPA), the existence of morbidity, patients comorbidity (CCI) and hospital stay were significant risk factors associated with costs > US$7500 in the univariate analysis. Only morbidity and hospital stay were shown to be significant independent risk factors in the multivariable analysis (*P* < 0.0001 and *P* = 0.0436 respectively).

**Table 3:** Factors affecting treatment costs: uni- and multivariable logistic regression analysis

	*n* patients	Costs > US$7500	Risk for costs > US$7500			
			Univariable analysis		Multivariable analysis	
			OR (95% CI)	*P* value	OR (95% CI)	*P* value
Sex						
Women	63	19	0.182 (− 0.301 to 0.679)	0.721	Not included in the model	NC
Men	23	10	1.00			
Laterality						
Left	49	16	0.389 (0.089 to 1.718)	0.189	Not included in the model	NC
Right	37	13	1.00			
Surgical technique						
LTA	43	20	1.276 (0.787 to 2.090)	0.013^‡^	2.522 (0.479 to 12.04)	0.312
RPA	43	9	1.00		1.00	
Resection						
Partial	12	5	1.451 (0.893 to 2.351)	0.532	Not included in the model	NC
Total	74	24	1.00			
Tumor size (mm)						
> 40	34	11	0.751 (0.183 to 3.201)	0.692	Not included in the model	NC
≤ 40	52	18	1.00			
Morbidity						
Yes	35	26	34.1 (9.438 to 150.7)	< 0.0001^‡^	33.07 (8.003 to 185.5)	< 0.0001^‡^
No	51	3	1.00		1.00	
BMI (kg/m^2^)						
> 25	22	16	2.083 (0.707 to 5.988)	0.173	Not included in the model	NC
≤ 25	64	13	1.00			
Hormonally active tumor						
Yes	56	18	0.886 (0.543 to 1.436)	0.0877	Not included in the model	NC
No	30	11	1.00			
Prior abdo. surgery						
Yes	17	9	1.003 (0.345 to 3.476)	0.480	Not included in the model	NC
No	69	20	1.00			
CCI						
> 2	29	20	3.827 (1.351 to 12.02)	0.0147^‡^	1.440 (0.309 to 7.011)	0.638
≤ 2	57	9	1.00		1.00	
Hospital stay (days)						
> 5	30	27	7.500 (1.952 to 45.59)	0.0103^‡^	7.344 (1.278 to 69.27)	0.0436^‡^
≤ 5	56	2	1.00		1.00	

*LTA* lateral transperitoneal adrenalectomy, *RPA* retroperitoneoscopic adrenalectomy, *BMI* body mass index, *OR* odds ratio, *CI* confidence interval, *CCI* Charlson Comorbidity Index, *NC* not calculated

^‡^Statistically significant

### Factors influencing the profit of both surgical procedures

Similar to the cost analysis, an analysis of the influencing factors for financial loss was carried out by means of uni- and multivariable logistic regression (Table 4). The existence of morbidity, patients comorbidity (CCI) and hospital stay were significant risk factors associated with financial loss in the univariate analysis. Only morbidity and hospital stay were shown to be significant independent risk factors in the multivariable analysis (*P* < 0.0001 and *P* = 0.021, respectively).

**Table 4:** Factors affecting treatment profit: uni- and multivariable logistic regression

	*n* patients	Profit < US$0	Risk for profit < US$0			
			Univariable analysis		Multivariable analysis	
			OR (95% CI)	*P* value	OR (95% CI)	*P* value
Sex						
Women	63	14	0.571 (0.209–1.524)	0.271	Not included in the model	NC
Men	23	10	1.00			
Laterality						
Left	49	11	0.403 (0.090–1.698)	0.383	Not included in the model	NC
Right	37	13	1.00			
Surgical technique						
LTA	43	17	1.200 (0.518–2.843)	0.053	1.265 (0.646–10.65)	0.206
RPA	43	7	1.00			
Resection						
Partial	12	6	0.531 (0.032–2.312)	0.619	Not included in the model	NC
Total	74	18	1.00			
Tumor size (mm)						
> 40 mm	34	10	1.219 (0.687–2.139)	0.731	Not included in the model	NC
≤ 40 mm	52	14	1.00			
Morbidity						
Yes	35	19	17.5 (7.493–43.81)	< 0.0001^‡^	16.32 (7.092–44.23)	< 0.0001^‡^
No	51	5	1.00			
BMI (kg/m^2^)						
> 25	22	7	1.126 (0.395–3.532)	0.829	Not included in the model	NC
≤ 25	64	17	1.00			
Hormonal active tumor						
Yes	56	14	1.143 (0.435–3.876)	0.489	Not included in the model	NC
No	30	10	1.00			
Prior abdo. surgery						
Yes	17	8	1.091 (0.531–3.641)	0.489	Not included in the model	NC
No	69	16	1.00			
CCI						
> 2	29	15	2.143 (0.903–5.611)	0.0394^‡^	1.765 (0.724–5.011)	0.151
≤ 2	57	9	1.00			
Hospital stay (days)						
> 5	30	16	6.316 (2.238–19.96)	0.008^‡^	6.036 (2.119–19.97)	0.021^‡^
≤ 5	56	8	1.00			

*LTA* lateral transperitoneal adrenalectomy, *RPA* retroperitoneoscopic adrenalectomy, *BMI* body mass index, *OR* odds ratio, *CI* confidence interval, *CCI* Charlson Comorbidity Index, *NC* not calculated

^‡^Statistically significant

## Discussion

Analyzing our matched cohorts of patients with various adrenal pathologies, RPA conferred superior costs/profit profile compared to LTA. This superiority of RPA may be associated with a lower risk of perioperative complications, a reduced use of postoperative drugs and reduced hospital stay.

Several studies have examined the economic costs associated with adrenal surgery. For example, surgical treatment of Conn syndrome results in a saving of $31,132 for the healthcare system over the lifetime of the patient when compared to conservative medical treatment [41, 42]. Moreover, laparoscopic surgery significantly reduces hospital costs further (17.9% reduction in total hospital costs) when compared to the costs resulting from open surgery [43]. While the laparoscopic technique is associated with an 18.1% increase in intraoperative costs this is offset by the postoperative costs which are 63.4% lower [44]. The use of surgeons with extensive experience in adrenal surgery results in an additional reduction in overall costs [45–47].

Much less is known about the economic impact of RTA versus LTA surgery, with the published data to date being sparse and often seemingly contradictory [48, 49]. Moreover, many studies have limited their analysis to direct surgical costs and have exclusively focussed on data from the US health care system [50]. In fact, there are no data directly comparing costs, revenues and possible profit associated with these two surgical approaches. Given the significant differences between the costs, funding and reimbursement in the US healthcare system versus European healthcare models, it is difficult to extrapolate and draw firm conclusions from the existing data.

Therefore, we sought to perform a comprehensive cost–benefit analysis directly comparing LTA and RTA in patients who underwent adrenalectomy using a matched-case study design.

Both cohorts (*n* = 43) were similar in terms of demographic and clinical parameters. Although the revenue for LTA tended to be slightly higher (US$7244 vs. v US$7204) than for the RPA, this difference was not statistically significant (*P* = 0.487). This total revenue difference between LTA and RPA can be explained by the supplements and deductions specific to gDRG system. It should be noted that baseline revenue calculation was based on the German DRG system, which makes no difference between RPA and LTA in terms of the type of procedure, both procedures being coded as minimally invasive adrenalectomy.

In terms of overall costs, LTA is more expensive than RPA (US$6618 vs. US$5790 and US$5512 vs. US$5006 for mean and median values respectively). This difference is statistically significant (*P* = 0.043). The costs were generated by the same factors in both procedures, namely (in order of decreasing share) (1) the nursing costs on a general surgical ward, (2) the ward infrastructure, (3) medical expenses in the operating room and (4) physician-associated cost. The detailed analysis of cost centers revealed that the general surgical ward was responsible for most of the costs, followed by the operating room and the anesthestics (induction and recovery rooms). These are similar in any surgical department and were consistent between both groups (RPA vs. LTA). Medical needs in the OR and OR infrastructure differed significantly in both groups. The cost center “infrastructure operating room” consists in part of the costs associated with the use of the operating room and the instruments available on that facilites. As the operating time is statistically longer in LTA than in the RPA group, this explains why the cost center “infrastructure operating room” is higher in the LTA than in the RPA group. The leading cost bearers (with decreasing total share) were (1) physicians, (2) infrastructure, (3) nursing. Previous studies have shown that personnel costs, in the context of surgical treatments, account for the majority of the expenditure [51, 52]. This remains the case for minimally invasive adrenal procedure. We hypothesize that the benefits of RPA over LTA in terms of duration of surgery, postoperative morbidity, and reduced postoperative drug use (benefits found both in our series and in the international literature) account for the cost difference between the two techniques.

However, our analysis revealed that both techniques differed significantly in terms of profit. The median profit and the mean profit were statistically significantly different between both cohorts (*P* = 0.027). RPA led to a significantly higher profit than LTA (US$1235 vs. US$653 and US$1296 vs. US$789 respectively for mean and median profit). This finding is new and contradicts current studies [48–50], which in most cases failed to show any difference between these procedures. It should be noted here that most of the studies were purely cost-related, and predominantly restricted their analyses to operational costs. A full economic costing, taking into all of the key stakeholders and factors into account has been lacking.

**Fig. 2:**
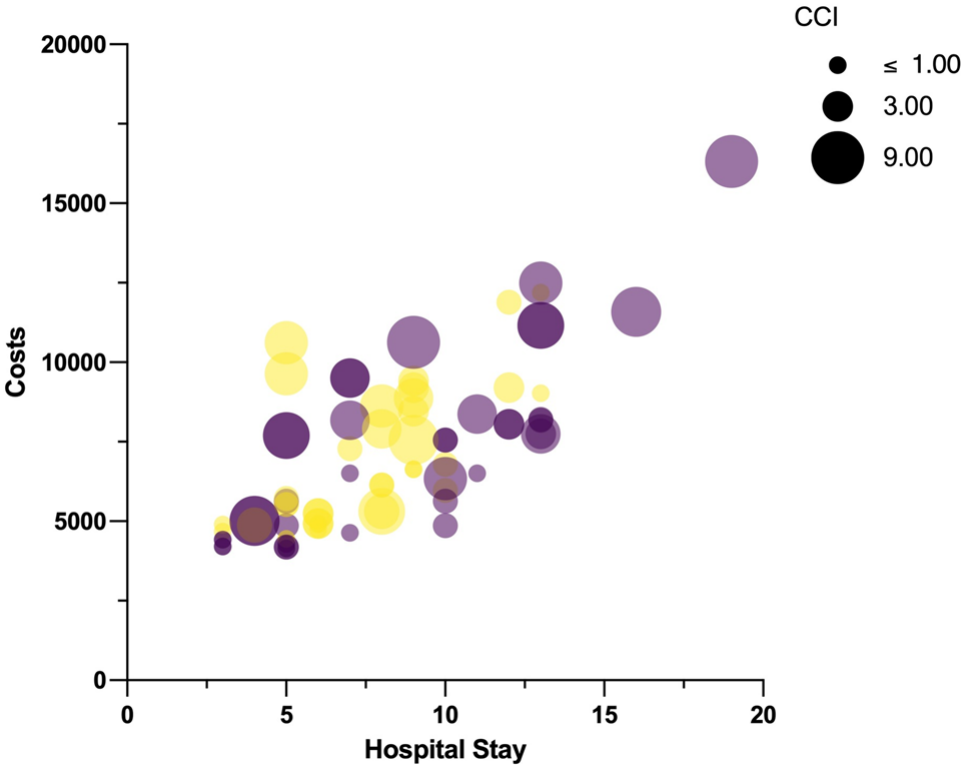
Multidimensional cost-analysis (US$) according to hospital stay, comorbidity and procedure. Length of hospital stay is represented in (*x*) coordinate (abscissa), costs are represented in (*y*) coordinate (ordinate). Each bubble corresponds to a patient and the size of the bubble is directly proportional to the CCI value of that patient (comorbidity). Yellow bubble: *RPA* retroperitoneoscopic adrenalectomy; violet bubble: *LTA* lateral transperitoneal adrenalectomy (Color figure online)

The multivariate logistic regression analysis showed that the factors influencing treatment costs and profit (Tables 3, 4; Fig. 2) were the duration of hospitalization (*P* = 0.0436 and *P* = 0.021 respectively) and postoperative morbidity (*P* < 0.0001). No other significant factors were identified in this analysis. This result is consistent with the published literature [53–55]. Although surgical technique was not identified as significant factor in the multivariate analysis, it should be kept in mind that RPA was associated with significantly less overall morbidity and a shorter period of hospitalization. Thus, it is at least conceivable that the procedure itself may significantly impact upon costs and profit in larger randomized cohorts.

The large number of postoperative complications registered in our series is due to the method of accounting for morbidity. Any deviation from the optimal postoperative trajectory was coded as morbidity. Thus, a simple anomaly in the biological results was interpreted as such. In addition, we considered all events, as one patient may have several postoperative complications. This explains why the total number is large compared to other series dealing with minimally invasive surgery of the adrenal gland.

In our series, the calculation of revenue and profit is based on the gDRG system. In 2011, German hospital financing was changed to the gDRG, which is based on the US-DRG system. There, Robert B. Fetter of Yale University had already proposed in 1967 that the financing of hospital costs should be based on prospectively determined and disease-specific case financing. In the USA, this system was used since the early 1980s. Subsequently, it was implemented in Australia. The diagnosis-related group systems in Europe (EuroDRG project) brings together 12 countries: Austria, England, Estonia, Finland, France, Germany, Ireland, the Netherlands, Poland, Portugal, Spain and Sweden.

This study has a number of limitations. Our investigation may be subject to inherent biases based on its pair-matching and monocentric nature. Selection bias may have resulted in patients with more complex comorbidities being over- or underrepresented in one particular group. Another limitation of the study is that our database does not offer data on the expertise of individual surgeons for both techniques.

In conclusion, RPA conferred superior cost/profit profile associated with a lower risk of perioperative complications and hospital stay compared to LTA in a matched cohort of patients with various adrenal pathologies.

### Supplementary Information

Below is the link to the electronic supplementary material.

Supplementary file1 (DOCX 31 kb)

Supplementary file2 (DOCX 25 kb)

## Data Availability

Data are available on request.
